# Oral Zinc Supplementation for the Treatment of Acute Diarrhea in Children: A Systematic Review and Meta-Analysis

**DOI:** 10.3390/nu5114715

**Published:** 2013-11-21

**Authors:** Laura M. Lamberti, Christa L. Fischer Walker, Kit Y. Chan, Wei-Yan Jian, Robert E. Black

**Affiliations:** 1 Department of International Health, Johns Hopkins Bloomberg School of Public Health, 615 N. Wolfe St, Baltimore, MD 21205, USA; E-Mails: llambert@jhsph.edu (L.M.L.); rblack@jhsph.edu (R.E.B.); 2 Department of Health Policy and Management, School of Public Health, Peking University Health Science Centre, 38 Xueyuan Rd. in Haidian District, Beijing 10083, China; E-Mails: k.chan@ed.ac.uk (K.Y.C.); jianweiyan@bjmu.edu.cn (W.-Y.J.); 3 Centre for Population Health Sciences, University of Edinburgh Medical School, Teviot Place, Edinburgh, Scotland EH8 9AG, UK

**Keywords:** zinc, children, global health, China

## Abstract

Evidence supporting the impact of therapeutic zinc supplementation on the duration and severity of diarrhea among children under five is largely derived from studies conducted in South Asia. China experiences a substantial portion of the global burden of diarrhea, but the impact of zinc treatment among children under five has not been well documented by previously published systematic reviews on the topic. We therefore conducted a systematic literature review, which included an exhaustive search of the Chinese literature, in an effort to update previously published estimates of the effect of therapeutic zinc. We conducted systematic literature searches in various databases, including the China National Knowledge Infrastructure (CNKI), and abstracted relevant data from studies meeting our inclusion and exclusion criteria. We used STATA 12.0 to pool select outcomes and to generate estimates of percentage difference and relative risk comparing outcomes between zinc and control groups. We identified 89 Chinese and 15 non-Chinese studies for the review, including studies in 10 countries from all WHO geographic regions, and analyzed a total of 18,822 diarrhea cases (9469 zinc and 9353 control). None of the included Chinese studies had previously been included in published pooled effect estimates. Chinese and non-Chinese studies reported the effect of therapeutic zinc supplementation on decreased episode duration, stool output, stool frequency, hospitalization duration and proportion of episodes lasting beyond three and seven days. Pooling Chinese and non-Chinese studies yielded an overall 26% (95% CI: 20%−32%) reduction in the estimated relative risk of diarrhea lasting beyond three days among zinc-treated children. Studies conducted in and outside China report reductions in morbidity as a result of oral therapeutic zinc supplementation for acute diarrhea among children under five years of age. The WHO recommendation for zinc treatment of diarrhea episodes should be supported in all low- and middle-income countries.

## 1. Introduction

In response to mounting evidence supporting the efficacy and effectiveness of therapeutic zinc supplementation for diarrhea among children under five years of age, the World Health Organization (WHO) and the United Nation’s Children Fund (UNICEF) issued a global recommendation in 2004, which advised zinc supplementation in addition to oral rehydration solution (ORS) for the treatment of all diarrhea episodes among children <5 years of age [1,2]. Systematic reviews have quantified the association between therapeutic zinc supplementation and a reduction in the duration and severity of childhood diarrhea episodes in low- and middle-income countries (LMICs) [1,3,4]. Many of the studies contributing to this body of evidence were conducted in South Asia [5,6,7], but literature stemming from East Asia has not been included in past reviews. In 2011, Zhang published a systematic review which identified 11 Chinese studies assessing zinc treatment for diarrhea and signified the need to update previous meta-analyses with literature published in languages other than English [8].

We sought to conduct an extensive search for studies of oral therapeutic zinc supplementation published in Chinese and any other language. We also aimed to combine evidence across regions in order to generate global estimates of the effect of oral therapeutic zinc supplementation on selected morbidity and mortality outcomes among children under five years of age.

## 2. Methods

We conducted a systematic literature search for studies published in any language between 1980 and November 2012 using the MeSH search terms “zinc” and “diarrhea” limited to “humans” in the following databases: Biosis, Cumulative Index to Nursing and Allied Health (CINAHL), Cochrane Central Register of Controlled Trials (CENTRAL), Embase, the WHO International Clinical Trials Registry Platform (ICTRP), Global Health, Latin American and Caribbean Health Sciences Literature (LILACS), PubMed, Scopus, Web of Science, IndMed, Egyptian Universities Library Consortium, Index Medicus for the Eastern Mediterranean Region (IMEMR), China National Knowledge Infrastructure (CNKI), WanFang, and Chinese BioMedical (CBM) database.

Titles and abstracts were reviewed by two independent reviewers, and complete manuscripts were obtained for further review of pertinent studies. Discrepancies were resolved in consultation with a third reviewer. We restricted inclusion to individually randomized controlled trials (RCTs) of children under five years of age with acute diarrhea, including dysentery, where diarrhea was defined as the passage of at least three loose or watery stools in a 24-h period. We excluded cluster RCTs, studies that exclusively enrolled a particular subgroup of children (e.g., HIV-infected children; preterm infants), and studies of persistent diarrhea. We included RCTs assessing oral zinc supplementation of any zinc salt in comparison to a control group receiving placebo supplement. For studies conducted in China, where placebo supplements may not have been readily available, we included trials in which cases received the same supportive therapy regardless of zinc allocation. For all studies, administration of minerals (excluding iron), vitamins, and supporting therapy beyond zinc were only considered acceptable if these were received by both the intervention and control groups. Studies that used supplements that included iron, zinc-fortified ORS, or zinc-fortified foods were excluded.

Included studies were reviewed for the following outcomes: diarrhea duration; the proportion of diarrhea episodes lasting >3 and >7 days; duration of hospitalization; duration of fever; duration of vomiting; proportion of cases vomiting; stool frequency (number per day); stool output (mL); and death from diarrhea or any cause. Two independent reviewers entered data into structured tables, and discrepancies were resolved in consultation with a third reviewer.

We conducted independent analyses for studies assessing diarrhea due to unspecified causes and those assessing specific pathogens (e.g., rotavirus) that were laboratory confirmed prior to enrollment. All data analyses were conducted in STATA 12.0 [9]. We fit Poisson and logistic regression models to continuous and binary outcomes, respectively, weighting all outcomes by sample size. These models generated pooled estimates and 95% confidence intervals lower bound by zero for all outcomes and upper bound by one for proportions.

For continuous outcomes, we calculated the overall percentage difference between the pooled estimates for the zinc and control groups. For binary outcomes, we calculated estimates of relative risk (RR) with placebo as the reference group and conducted random effects meta-analyses to combine RRs across studies [9].

We conducted hypothesis testing to assess the equivalence of pooled outcomes and of effect estimates by placebo and non-placebo controlled trials. To compare effect estimates, we tested the difference of mean percentage differences for continuous outcomes and the ratio of relative risks (RRR) for binary outcomes [10]. We subsequently pooled placebo and non-placebo controlled trials for outcomes with no statistically significant difference in effect size.

We assessed the association between the dose of oral zinc supplement and diarrhea duration by regressing the mean percentage difference in diarrhea duration comparing the zinc and control groups onto a categorical variable which indicated whether zinc dose was lower than, equal to, or greater than the WHO recommendation.

During the course of our analyses, we identified a zinc product called Licorzinc that appeared to be unique to China. To determine whether outcomes for Chinese studies were generalizable comparing Licorzinc to other better established zinc products, we conducted hypothesis testing to assess the equivalence of the mean percentage difference in episode duration between zinc and placebo. We also calculated the RRR to compare the RR of episodes lasting >3 days between studies using Licorzinc and other zinc products.

We plotted funnel plots to assess our primary outcomes for publication bias. We also employed the Child Health Epidemiology Reference Group (CHERG) grading system to assess the quality of evidence for each outcome on a four-point scale (“high”, “moderate”, “low”, “very low”) [11].

## 3. Results

**Figure 1:**
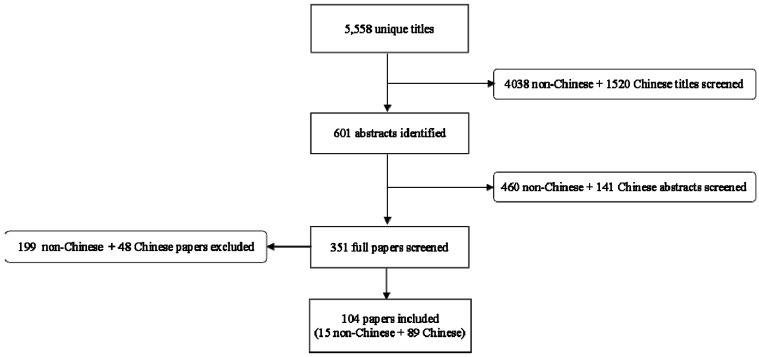
Results of systematic literature search and review.

The systematic literature search of the non-Chinese databases uncovered 4038 titles, and 15 were included after subsequent review of abstracts and full manuscripts for inclusion and exclusion criteria (Figure 1) [5,6,7,12,13,14,15,16,17,18,19,20,21,22,23]. Of the included studies, 13 were conducted in a hospital setting and two assessed episodes occurring in the community. Included studies were conducted in sites located within 10 countries: India (*n* = 6); Bangladesh (*n* = 5); Nepal (*n* = 1); Turkey (*n* = 1); Brazil (*n* = 1); Pakistan (*n* = 1); Ethiopia (*n* = 1); Yemen (*n* = 1); and Poland (*n* = 1). These studies enrolled a total of 3271 zinc-allocated and 3314 placebo-allocated diarrhea cases. The systematic literature search for Chinese studies resulted in 1520 titles, of which 89 were included (Figure 1) [24,25,26,27,28,29,30,31,32,33,34,35,36,37,38,39,40,41,42,43,44,45,46,47,48,49,50,51,52,53,54,55,56,57,58,59,60,61,62,63,64,65,66,67,68,69,70,71,72,73,74,75,76,77,78,79,80,81,82,83,84,85,86,87,88,89,90,91,92,93,94,95,96,97,98,99,100,101,102,103,104,105,106,107,108,109,110,111,112]. All included studies were conducted in a hospital setting, and 33 studies focused on diarrhea attributable to laboratory confirmed rotavirus. None of the included studies identified through the Chinese database were placebo-controlled; for Chinese studies, zinc and control groups received a range of supportive treatments, including fluid infusion, probiotics and antivirals. The total enrolment of included Chinese studies was 6198 zinc group and 6039 control group diarrhea cases. Table 1 describes the trial setting, sample size, and zinc intervention for all included studies.

**Table 1:** Characteristics of included studies.

Author [Reference]	Year Published	Country	Trial Setting	Specific Causative Organisms	Age Group (months)	Sample Size		Zinc Salt	Tablet or Syrup	Daily Zinc Dose	Length of Supplementation (days)
						Zinc Group	Control Group				
Al Sonboli [17]	2003	Brazil	Hospital	Unknown	3–60	37	37	Not Listed	Tablet	3–5 mos: 22.5 mg 6–60 mos: 45 mg	5
Bahl [7]	2002	India	Community	Unknown	6–35	404	401	Zinc Gluconate	Syrup	6–11 mos: 15 mg 12–35 mos: 30 mg	14
Brooks [16]	2005	Bangladesh	Hospital	Unknown	1–6	91	93	Zinc Acetate	Syrup	20 mg	Duration of episode
Brooks [16]	2005	Bangladesh	Hospital	Unknown	1–6	91	93	Zinc Acetate	Syrup	5 mg	Duration of episode
Dutta [23]	2011	India	Hospital	Unknown	6–24	44	41	Not Listed	Syrup	40 mg	14
Elnemr [21]	2007	Yemen	Hospital	Unknown	3–24	88	92	Zinc Acetate	Syrup	20 mg	14
Faruque [12]	1999	Bangladesh	Hospital	Unknown	6–24	343	341	Zinc Acetate	Syrup	14.2 mg	15
Fischer Walker [19]	2006	Pakistan	Hospital	Unknown	1–5	281	279	Zinc Sulfate	Tablet	10 mg	14
Fischer Walker [19]	2006	India	Hospital	Unknown	1–5	186	187	Zinc Sulfate	Tablet	10 mg	14
Fischer Walker [19]	2006	Ethiopia	Hospital	Unknown	1–5	87	90	Zinc Sulfate	Tablet	10 mg	14
Larson [18]	2005	Bangladesh	Hospital	Unknown	3–59	267	266	Zinc Sulfate	Tablet	20 mg	10
Patel [20]	2009	India	Hospital	Unknown	6–59	264	271	Zinc Sulfate	Syrup	20 mg	14
Patro [22]	2010	Poland	Hospital	Unknown	3–48	81	79	Zinc Sulfate	Syrup	3–5 mos: 10 mg 6–48 mos: 20 mg	10
Polat [15]	2003	Turkey	Hospital	Unknown	2–29	52	54	Zinc Sulfate	Syrup	20 mg	10
Roy [13]	1999	Bangladesh	Hospital	Unknown	3–24	32	35	Zinc Acetate	Syrup	20 mg	14
Sachdev [5]	1988	India	Hospital	Unknown	6–18	25	25	Zinc Sulfate	Tablet	40 mg	Not Listed
Sazawal [6]	1995	India	Hospital	Unknown	6–35	456	481	Zinc Gluconate	Syrup	20 mg	Not Listed
Strand [14]	2002	Nepal	Community	Unknown	6–35	442	449	Not Listed	Syrup	6–11 mos: 15 mg 12–35 mos: 30 mg	From enrolment until 7 days after episode subsided
Zhao [24]	2011	China	Hospital	Unknown	4–36	40	40	Licorzinc	Tablet	4–5 mos: 10.8 mg 6–12 mos: 14.4 mg 13–36 mos: 21.6 mg	Not Listed
Zhang [25]	2009	China	Hospital	Rotavirus	6–24	60	60	Zinc Gluconate	Not Listed	20 mg	Duration of episode
Lin [26]	2010	China	Hospital	Rotavirus	1.5–36	58	58	Zinc Gluconate	Syrup	1.5–5 mos: 10 mg 6–36 mos: 20 mg	Duration of episode
Zhou [27]	2010	China	Hospital	Rotavirus	6–24	42	40	Zinc Gluconate	Not Listed	20 mg	14
Yang [28]	2011	China	Hospital	Unknown	3–36	42	40	Zinc Gluconate	Tablet	3–5 mos: 10 mg 6–36 mos: 20 mg	10–14
Liu [29]	2010	China	Hospital	Unknown	5–18	40	40	Zinc Gluconate	Not Listed	5 mos: 10 mg 6–18 mos: 20 mg	10–14
Chen [30]	2006	China	Hospital	Rotavirus	0–24	30	30	Zinc gluconate	Not Listed	10 mg	Not Listed
Liu [31]	2011	China	Hospital	Unknown	6.8–22	90	90	Zinc Gluconate	Tablet	20 mg	Not Listed
Liu [32]	2009	China	Hospital	Unknown	6–36	112	108	Zinc Gluconate	Tablet	20 mg	10
Fu [33]	2010	China	Hospital	Rotavirus	2–24	98	102	Zinc Gluconate	Syrup	5 mg	Not Listed
Zhou [34]	2008	China	Hospital	Unknown	2–48	40	40	Licorzinc	Not Listed	2–5 mos: 7.5 mg 6–12 mos: 11.25 mg 13–48 mos: 18.75 mg	10–14
Chen [35]	2008	China	Hospital	Rotavirus	4–48	60	60	Licorzinc	Not Listed	4–5 mos: 7.2 mg 6–48 mos: 10.8 mg	Not Listed
Guan [36]	2012	China	Hospital	Rotavirus	1.5–45.6	45	45	Licorzinc	Not Listed	1.5–5 mos: 7.5 mg 6–11 mos: 11.25 mg 12–45.6 mos: 18.75 mg	10–14
Wu [37]	2010	China	Hospital	Rotavirus	4–13	46	46	Licorzinc	Not Listed	4–5 mos: 10 mg 6–13 mos: 20 mg	Not Listed
Zhou [38]	2010	China	Hospital	Unknown	6–24	65	60	Licorzinc	Tablet	20 mg	Not Listed
Luo [39]	2009	China	Hospital	rotavirus	6–36	55	50	Licorzinc	Tablet	18.75 mg	Not Listed
Zhang [40]	2010	China	Hospital	Unknown	5–48	50	50	Licorzinc	Not Listed	Not Listed *	Not Listed
Ju [41]	2007	China	Hospital	Unknown	6–36	40	38	Licorzinc	Tablet	6–12 mos: 11–25 mg 13–36 mos: 15 mg	Not Listed
Wang [42]	2012	China	Hospital	Unknown	6–36	30	30	Licorzinc	Tablet	Not Listed *	3
Hong [43]	2009	China	Hospital	Rotavirus	3–60	140	120	Zinc Sulfate	Syrup	3–11 mos: 20 mg 12–36 mos: 30 mg 37–60 mos: 40 mg	Not Listed
Lin [44]	1994	China	Hospital	Unknown	0.5–24	46	58	Zinc Sulfate	Syrup	10–14 mg/kg *	Not Listed
Yan [45]	2011	China	Hospital	Unknown	5–36	70	50	Zinc Sulfate	Syrup	5 mos: 50 mg 6–36 mos: 100 mg	Not Listed
He [46]	1997	China	Hospital	Unknown	6–36	52	58	Zinc Gluconate	Not Listed	20 mg	Not Listed
Wei [47]	2011	China	Hospital	Unknown	3–36	44	42	Zinc Gluconate	Syrup	3–5 mos: 10 mg 6–36 mos: 20 mg	10–14
Yang [48]	2012	China	Hospital	Unknown	0–36	80	80	Zinc Gluconate	Tablet	0–5 mos: 10 mg 6–36 mos: 20 mg	10
Pu [49]	2010	China	Hospital	Rotavirus	0–24	38	34	Zinc Gluconate	Not Listed	0–5 mos: 10 mg 6–24 mos: 20 mg	Not Listed
Zhang [50]	2011	China	Hospital	Rotavirus	3–36	53	53	Zinc Gluconate	Not Listed	3–5 mos: 10 mg 6–36 mos: 20 mg	10
Sun [51]	2008	China	Hospital	Unknown	1.5–36	45	45	Zinc Gluconate	Syrup	1.5–5 mos: 10 mg 6–36 mos: 20 mg	Not Listed
Zhang [52]	2011	China	Hospital	Unknown	3–36	90	90	Zinc Gluconate	Syrup	3–5 mos: 10 mg 6–36 mos: 20 mg	Not Listed
Lin [53]	2010	China	Hospital	Rotavirus	6–54	28	20	Zinc Gluconate	Tablet	6–54 mos: 20 mg	14
Liu [54]	2009	China	Hospital	Unknown	3–36	95	91	Zinc Gluconate	Not Listed	3–5 mos: 10 mg 6–36 mos: 20 mg	10–14
Qiao [55]	2011	China	Hospital	Unknown	6–36	73	72	Zinc Gluconate	Tablet	6–36 mos: 20 mg	14
Zhang [56]	2007	China	Hospital	Unknown	0–24	85	90	Zinc Gluconate	Not Listed	0–5 mos: 10 mg 6–24 mos: 20 mg	10
Zhao [57]	2012	China	Hospital	Unknown	0–24	70	70	Zinc Gluconate	Syrup	0–5 mos: 10 mg 6–24 mos: 20 mg	10–14
Cai [58]	2011	China	Hospital	Unknown	0–24	88	84	Zinc Gluconate	Not Listed	0–5 mos: 10 mg 6–24 mos: 20 mg	14
Zhang [59]	2012	China	Hospital	Rotavirus	6–17	120	120	Zinc Gluconate	Tablet	20 mg	10–14
Qiao [60]	2012	China	Hospital	Unknown	0–24	85	85	Zinc Gluconate	Not Listed	0–5 mos: 10 mg 6–24 mos: 20 mg	10
Zhong [61]	2012	China	Hospital	Rotavirus	3–48	50	50	Zinc Gluconate	Tablet	3–5 mos: 10 mg 6–48 mos: 20 mg	10
Wang [62]	2011	China	Hospital	Rotavirus	0–24	60	60	Zinc Gluconate	Not Listed	0–5 mos: 10 mg 6–24 mos: 20 mg	10
Yang [63]	2008	China	Hospital	Rotavirus	0–36	164	168	Zinc Gluconate	Not Listed	0–5 mos: 10 mg 6–36 mos: 20 mg	10
Zhao [64]	2012	China	Hospital	Rotavirus	6–36	60	60	Zinc Gluconate	Syrup	35 mg	10
Ma [65]	2012	China	Hospital	Rotavirus	4–42	41	41	Zinc Gluconate	Not Listed	20 mg	Not Listed
Chen [66]	2012	China	Hospital	Rotavirus	0–36	93	93	Zinc Gluconate	Not Listed	0–5 mos: 10 mg 6–36 mos: 20 mg	10
Hu [67]	2009	China	Hospital	Rotavirus	4–36	60	60	Zinc Gluconate	Tablet	4–5 mos: 10 mg 6–36 mos: 20 mg	10
Yuan [68]	2011	China	Hospital	Unknown	1–36	100	100	Zinc Gluconate	Tablet	1–12 mos: 70 mg 13–36 mos: 140 mg	14
Tan [69]	2011	China	Hospital	Unknown	3–36	50	35	Zinc Gluconate	Tablet	3–5 mos: 10 mg 6–36 mos: 20 mg	10–14
Liu [70]	2010	China	Hospital	Unknown	0–36	89	77	Zinc Gluconate	Syrup	0–5 mos: 10 mg 6–36 mos: 20 mg	10
Hu [71]	2011	China	Hospital	Unknown	3–60	108	100	Zinc Gluconate	Tablet	3–5 mos: 10 mg 6–60 mos: 20 mg	14
Li [72]	2008	China	Hospital	Unknown	6–36	40	38	Zinc Gluconate	Tablet	6–12 mos: 7.5 mg 13–36 mos: 15 mg	3
Gao [73]	2012	China	Hospital	Unknown	3–36	74	74	Zinc Gluconate	Not Listed	3–5 mos: 10 mg 6–36 mos: 20 mg	14
Wu [74]	2011	China	Hospital	Unknown	3–60	20	20	Zinc Sulfate	Syrup	10 mg	10
Wu [74]	2011	China	Hospital	Unknown	3–60	20	20	Zinc Sulfate	Not Listed	10 mg	10
Liu [75]	2011	China	Hospital	Unknown	3–60	54	53	Zinc Gluconate	Tablet	3–5 mos: 10 mg 6–60 mos: 20 mg	3–5
Chen [76]	2010	China	Hospital	Unknown	5–36	42	20	Zinc Gluconate	Not Listed	5 mos: 10 mg 6–36 mos: 20 mg	10–14
Ma [77]	2012	China	Hospital	Unknown	2–36	63	63	Zinc Gluconate	Not Listed	2–5 mos: 70 mg 6–36 mos: 140 mg	10–14
Lu [78]	2012	China	Hospital	Unknown	6–18	120	140	Zinc Gluconate	Not Listed	140 mg	10–14
Ma [79]	2012	China	Hospital	Unknown	6–36	58	52	Zinc Gluconate	Syrup	6–36 mos: 20 mg	10
Ao [80]	2012	China	Hospital	Rotavirus	0–24	87	80	Zinc Gluconate	Syrup	0–5 mos: 10 mg 6–24 mos: 20 mg	Not Listed
Gu [81]	2011	China	Hospital	Unknown	3–60	56	60	Zinc Gluconate	Syrup	3–5 mos: 10 mg 6–60 mos: 20 mg	10
Wen [82]	2006	China	Hospital	Unknown	0–24	30	29	Zinc Gluconate	Not Listed	20 mg	10–14
Wang [83]	2011	China	Hospital	Unknown	3–36	60	60	Licorzinc	Not Listed	10–20 mg *	Duration of episode
Liu [84]	2012	China	Hospital	Rotavirus	8–30	90	90	Licorzinc	Not Listed	8–30 mos: 20 mg	Not Listed
Liu [85]	2012	China	Hospital	Unknown	3–60	100	100	Licorzinc	Tablet	3–5 mos: 10 mg 6–60 mos: 20 mg	Not Listed
Tong [86]	2011	China	Hospital	Unknown	2–36	98	98	Licorzinc	Not Listed	2–5 mos: 10 mg 6–36 mos: 20 mg	Not Listed
Qiu [87]	2010	China	Hospital	Rotavirus	1–24	53	52	Licorzinc	Tablet	1–5 mos: 10 mg 6–24 mos: 20 mg	14
Kong [88]	2011	China	Hospital	Unknown	3–30	35	35	Zinc Gluconate	Tablet	3–5 mos: 10 mg 6–11 mos: 15 mg 12–30 mos: 20 mg	14
He [89]	2007	China	Hospital	Rotavirus	5–22	60	63	Zinc Gluconate	Not Listed	20 mg	Not Listed
Kang [90]	2010	China	Hospital	Rotavirus	6–36	92	80	Zinc Gluconate	Tablet	20 mg	14
Su [91]	2012	China	Hospital	Rotavirus	6–36	97	97	Zinc Gluconate	Not Listed	20 mg	Not Listed
Huang [92]	2010	China	Hospital	Rotavirus	2–36	100	100	Not Listed	Tablet	2–5 mos: 10 mg 6–36 mos: 20 mg	Not Listed
Zhang [93]	2006	China	Hospital	Unknown	0–36	83	63	Licorzinc	Syrup	0–5 mos: 10 mg 6–36 mos: 20 mg	10–14
Wang [94]	2012	China	Hospital	Unknown	4–30	60	60	Zinc Gluconate	Syrup	10 mg	Not Listed
Lin [95]	2008	China	Hospital	Unknown	0.5–34	60	60	Zinc Gluconate	Tablet	0.5–5 mos: 140 mg 6–34 mos: 280 mg	10–14
Yan [96]	2011	China	Hospital	Unknown	6–60	57	57	Zinc Gluconate	Tablet	20 mg	10
Yu [97]	2012	China	Hospital	Unknown	0–36	40	40	Zinc Gluconate	Tablet	0–5 mos: 10 mg 6–36 mos: 20 mg	10–14
Zhang [98]	2011	China	Hospital	Rotavirus	4–36	128	128	Zinc Gluconate	Syrup	4–5 mos: 10 mg 6–36 mos: 20 mg	14
Xu [99]	2010	China	Hospital	Rotavirus	2–36	84	83	Zinc Gluconate	Not Listed	2–5 mos: 10 mg 6–36 mos: 20 mg	14
Tan [100]	2010	China	Hospital	Unknown	3.5–60	55	55	Zinc Gluconate	Syrup	3.5–5 mos: 10 mg 6–60 mos: 20 mg	10–14
Shen [101]	2012	China	Hospital	Rotavirus	2.5–40	46	42	Zinc Gluconate	Not Listed	2.5–5 mos: 10 mg 6–40 mos: 20 mg	Duration of episode
Wang [102]	2010	China	Hospital	Unknown	6–48	52	51	Zinc Gluconate	Tablet	20 mg	Not Listed
Chen [103]	2011	China	Hospital	Unknown	1–36	50	50	Zinc Gluconate	Tablet	1–5 mos: 5 mg 6–36 mos: 10 mg	Not Listed
Meng [104]	2012	China	Hospital	Unknown	0–24	90	90	Zinc Gluconate	Tablet	0–5 mos: 2.5 mg 6–12 mos: 5 mg 13–24 mos: 10 mg	Not Listed
Zhong [105]	2010	China	Hospital	Unknown	1–24	60	60	Zinc Gluconate	Tablet	1–5 mos: 2.5 mg 6–12 mos: 5 mg 13–24 mos: 7.5 mg	5–7
Xie [106]	2010	China	Hospital	Rotavirus	6–36	128	124	Zinc Gluconate	Tablet	20 mg	Not Listed
Fan [107]	2012	China	Hospital	Unknown	0–36	163	121	Not Listed	Not Listed	0–5 mos: 10 mg 6–36 mos: 20 mg	10
Zhou [108]	2012	China	Hospital	Rotavirus	6–24	75	75	Zinc Gluconate	Syrup	20 mg	10–14
Zhao [109]	2008	China	Hospital	Unknown	0–36	44	43	Zinc Gluconate	Tablet	0–5 mos: 10 mg 6–24 mos: 20 mg	Not Listed
Wan [110]	2006	China	Hospital	Unknown	6–36	26	24	Not Listed	Not Listed	Not Listed	Not Listed
Yang [111]	2012	China	Hospital	Unknown	6–60	60	60	Not Listed	Not Listed	20 mg	Not Listed
Luo [112]	2012	China	Hospital	Unknown	0–36	168	196	Not Listed	Not Listed	0–5 mos: 10 mg 6–36 mos: 20 mg	Not Listed

* Study not included in dose analyses.

The results of the studies identified through non-Chinese databases are summarized in Table 2 and Table 3. Acute episodes were 4% (95% CI: 1%–8%) shorter in duration among children treated with zinc compared to those receiving placebo (Table 2). Among children hospitalized for diarrhea, the duration of hospitalization was reduced by 37% (95% CI: 21%–53%) comparing the zinc and control groups (Table 2). Stool frequency was decreased by 6% (95% CI: 2%–10%) among zinc-treated children. Zinc-treated children had a reduced relative risk (RR) of acute diarrhea lasting beyond three and seven days and an increased risk of vomiting (RR: 1.83; 95% CI: 1.40–2.39) (Table 3).

**Table 2:** Pooled means of select outcomes for non-Chinese studies.

Outcome	Study Sites ^1^	Pooled Mean (95% CI) ^2^		Percent Difference ^3^
	*N*	Zinc Group	Control Group	(%)
**Duration of Episode (days)**	13	3.51 (3.43–3.60)	3.67 (3.59–3.76)	−4.4 (−7.8, −1.0)
**Duration of Hospitalization (days)**	1	2.00 (1.99–2.01)	3.17 (2.38–3.96)	−36.9 (−52.6, −21.2)
**Stool Output (mL)**	2	391.2 (388.5–393.8)	388.8 (386.2–391.5)	0.6 (−0.3, 1.6)
**Stool Frequency (Number per day)**	6	5.04 (4.88–5.19)	5.36 (5.20–5.52)	−6.0 (−9.9, −2.0)

^1^ Individual studies may contribute more than one study site (*N*) to each estimate; ^2^ Estimates for ≥2 study sites generated by Poisson regression model weighted by sample size; ^3^ Percent difference calculated by: 100 × ((Pooled Zinc Estimate − Pooled Control Estimate)/Pooled Control Estimate); 95% CI calculated by: Percent Difference ± 1.96 × {|(mean_zinc_/mean_control_)| × sqrt[(std error_zinc_)^2^/(mean_zinc_)^2^ + (std error_control_)^2^/(mean_control_)^2^]} × 100.

**Table 3:** Pooled relative risk of select outcomes for non-Chinese studies.

Outcome	Study Sites ^1^	Pooled Estimate Percentage (95% CI) ^2^		Pooled Relative Risk ^3^
	*N*	Zinc Group	Control Group	RR (95% CI)
**Episodes >** **3 days (%)**	3	29.7 (26.7–32.7)	39.5 (36.3–42.7)	0.78 (0.67–0.90)
**Episodes >** **7 days (%)**	6	10.3 (8.9–11.7)	14.9 (13.2–16.5)	0.74 (0.55–0.99)
**Vomiting (%)**	3	18.8 (16.0–21.6)	9.4 (7.3–11.4)	1.83 (1.40–2.39)

^1^ Individual studies may contribute more than one study site (*N*) to each estimate; ^2^ Estimates for ≥2 study sites generated by logistic regression model weighted by sample size; ^3^ Estimates for ≥2 studies generated by random effects meta-analysis.

**Figure 2:**
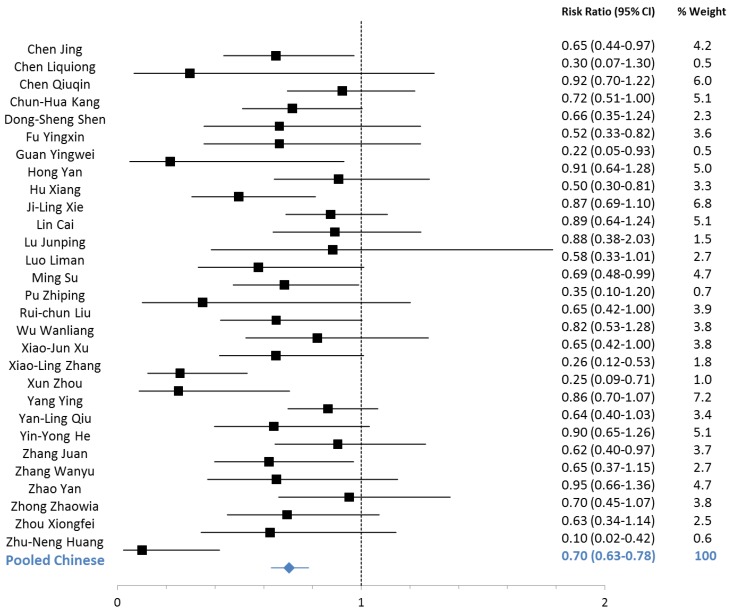
Forest plot for the effect of therapeutic zinc supplementation on Rotavirus diarrhea episodes >3 days.

**Figure 3:**
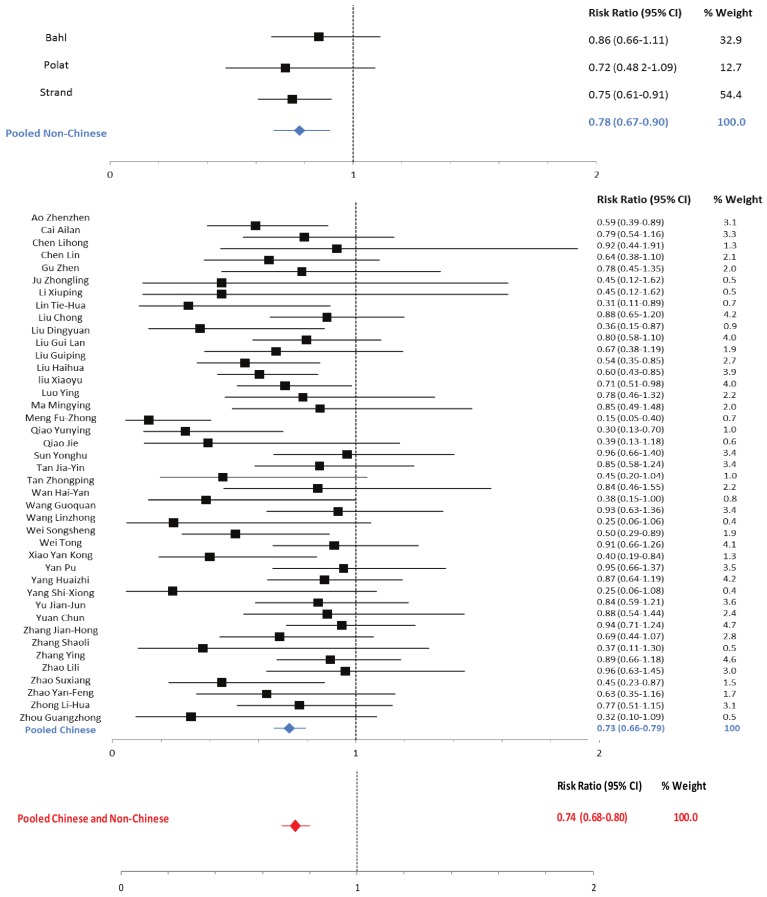
Forest plot for the effect of therapeutic zinc supplementation on non-specific diarrhea episodes lasting >3 days.

Outcomes pooled across studies conducted in China showed reductions in the duration of diarrhea, hospitalization, fever, vomiting, stool output and stool frequency among zinc-treated children with acute diarrhea attributable to rotavirus and to non-specific causes (Table 4). The reduction in the duration of diarrhea was 37% (95% CI: 35%–39%) among non-specific episodes and 31% (95% CI: 29%–34%) among rotavirus episodes (Table 4). The RR of diarrhea lasting beyond three days was reduced among zinc-treated patients with non-specific (RR: 0.73; 95% CI: 0.66–0.79) and rotavirus (RR: 0.70; 95% CI: 0.63–0.78) diarrhea (Table 5; Figure 2 and Figure 3).

**Table 4:** Pooled means of select outcomes for Chinese studies.

Outcome	Specific Causative Pathogens	Study Sites ^1^	Pooled Mean (95% CI) ^2^		Percent Difference ^3^
		*N*	Zinc Group	Control Group	(%)
**Duration of Episode (days)**	Unknown	40	2.96 (2.90–3.03)	4.68 (4.60–4.77)	−36.8 (−38.7, −34.8)
	Rotavirus	24	3.45 (3.36–3.54)	5.01 (4.89–5.12)	−31.1 (−33.5, −28.8)
**Duration of Hospitalization (days)**	Unknown	10	4.65 (4.50–4.80)	6.43 (6.25–6.61)	−27.7 (−30.8, −24.6)
	Rotavirus	2	4.15 (3.79–4.51)	6.1 (5.66–6.54)	−32.0 (−39.6, −24.3)
**Duration of** **Fever (days)**	Unknown	13	1.90 (1.80–1.99)	2.81 (2.70–2.92)	−32.4 (−36.5, −28.2)
	Rotavirus	4	1.96 (1.78–2.14)	3.18 (2.95–3.41)	−38.4 (−45.6, −31.2)
**Duration of Vomiting (days)**	Unknown	6	1.15 (1.05–1.25)	1.53 (1.41–1.64)	−24.8 (−33.3, −16.4)
	Rotavirus	3	1.84 (1.64–2.04)	2.49 (2.26–2.72)	−26.1 (−36.6, −15.6)
**Stool Output (mL)**	Unknown	1	40 (38.1–41.9)	70 (68.0–72.0)	−42.9 (−46.0, −39.7)
	Rotavirus	1	278.4 (256.8–300.0)	425.4 (382.1–468.7)	−34.6 (−42.9, −26.2)
**Stool Frequency (Number per day)**	Unknown	1	4 (3.8–4.2)	8 (7.6–8.4)	−50.0 (−53.5, −46.5)
	Rotavirus	2	3.74 (3.30–4.18)	4.27 (3.77–4.77)	−12.4 (−27.0, 2.1)

^1^ Individual studies may contribute more than one study site (*N*) to each estimate; ^2^ Estimates for ≥2 study sites generated by Poisson regression model weighted by sample size; ^3^ Percent difference calculated by: 100 × ((Pooled Zinc Estimate − Pooled Control Estimate)/Pooled Control Estimate); 95% CI calculated by: Percent Difference ± 1.96 × {|(mean_zinc_/mean_control_)| × sqrt[(std error_zinc_)^2^/(mean_zinc_)^2^ + (std error_control_)^2^/(mean_control_)^2^]} × 100.

**Table 5:** Pooled relative risk of select outcomes for Chinese studies.

Outcome	Specific Causative Pathogens	Study Sites ^1^	Pooled Estimate Percentage (95% CI) ^2^		Relative Risk ^3^
		*N*	Zinc Group	Control Group	RR (95% CI)
**Episodes >** **3 days (%)**	Unknown	44	31.4 (29.4–33.5)	49.2 (46.6–51.8)	0.73 (0.66–0.79)
	Rotavirus	29	31.8 (29.5–34.1)	50.3 (47.4–53.3)	0.70 (0.63–0.78)
**Episodes >** **7 days (%)**	Unknown	1	26.9 (-)	39.2 (-)	0.75 (0.42–1.37)

^1^ Individual studies may contribute more than one study site (*N*) to each estimate; ^2^ Estimates for ≥2 study sites generated by Poisson regression model weighted by sample size; ^3^ Estimates for ≥2 studies generated by random effects meta-analysis.

We did not identify any studies reporting diarrhea-specific or all-cause mortality for inclusion in this review. Nor did we identify non-Chinese studies reporting duration of fever or vomiting, or Chinese studies reporting the proportion of children vomiting.

The mean episode duration and proportion of episodes lasting >3 days were not statistically significantly different comparing zinc-treated children in Chinese and non-Chinese studies. There was no statistically significant difference between the estimated relative risk of an episode lasting >3 days (RRR: 1.07; 95% CI: 0.90–1.27) comparing Chinese and non-Chinese studies; therefore, we pooled this outcome across regions (RR: 0.74; 95% CI: 0.68–0.80) (Figure 3). The percentage difference between the mean episode duration of zinc-treated and control group children was statistically significantly larger for Chinese compared to non-Chinese studies (*p* < 0.05), so this outcome was not pooled across regions. We did not have sufficient power to compare other commonly reported outcomes by region.

Zinc dose was not associated with the mean percent difference in diarrhea duration comparing zinc and control children for non-Chinese (*p* = 0.50) or Chinese (*p* = 0.12) studies. Comparing Chinese studies that used Licorzinc to those that used other zinc supplements, there were no statistically significant differences in the mean percent difference in the duration of rotavirus episodes (*p* = 0.56), the RR of non-specific episodes lasting >3 days (RRR: 0.99; 95% CI: 0.72–1.35), or the RR of rotavirus episodes lasting >3 days (RRR: 0.93; 95% CI: 0.68–1.26). The percentage difference in the mean duration of non-specific episodes comparing zinc and control group children was statistically significantly higher for Licorzinc compared to “other zinc” studies (*p* = 0.01).

Our assessment of publication bias yielded largely symmetrical funnel plots for all outcomes.

Under the CHERG grading system, the studies included in this review were of moderate quality (Table 6) [11]. Effect estimates were largely consistent in directionality for all outcomes.

**Table 6:** Quality assessment of studies measuring the association between therapeutic zinc supplementation and selected outcomes.

Number of Studies	Design	Limitations	Consistency	Directness	
				Generalizability to Population of Interest	Generalizability to Intervention of Interest
***Diarrhea Duration (mean): Moderate outcome-specific quality*** ^1^					
53 non-specific 24 Rotavirus	RCT	Chinese studies not placebo-controlled (−0.5)	All but 4 studies showing decreased mean duration of diarrhea among zinc-treated children (+1)	Mostly South Asia and China (−0.5)	Generalizable
***Diarrhea Duration (>3 days): Moderate outcome-specific quality*** ^1^					
47 non-specific 29 Rotavirus	RCT	Chinese studies not placebo-controlled (−0.5)	All studies showing decreased risk of diarrhea duration >3 days among zinc-treated children (+1)	Mostly South Asia and China (−0.5)	Generalizable
***Diarrhea Duration (>7 days): Moderate outcome-specific quality*** ^1^					
7 non-specific	RCT	Chinese studies not placebo-controlled (−0.5)	All but one study showing decreased risk of diarrhea duration >7 days among zinc-treated children (+1)	Mostly South Asia and China (−0.5)	Generalizable
***Hospitalizations Duration: Moderate outcome-specific quality*** ^1^					
11 non-specific 2 Rotavirus	RCT	Chinese studies not placebo-controlled (−0.5)	All studies showing decreased mean duration of hospitalization among zinc-treated children (+1)	Only one non-Chinese study (−0.5)	Generalizable
***Stool Output: Moderate outcome-specific quality*** ^1^					
3 non-specific 1 Rotavirus	RCT	Chinese studies not placebo-controlled (−0.5)	All but one study showing decreased stool output among zinc-treated children (+1)	Only South Asia and China (−0.5)	Generalizable
***Stool Frequency: Moderate outcome-specific quality*** ^1^					
7 non-specific 2 Rotavirus	RCT	Chinese studies not placebo-controlled (−0.5)	All but three studies showing decreased stool frequency among zinc-treated children (+1)	Mostly South Asia and China (−0.5)	Generalizable
***Vomiting: Moderate outcome-specific quality*** ^1^					
3 non-specific	RCT	None	All studies showing increased vomiting among zinc-treated children (+1)	No Chinese studies (−0.5)	Generalizable
***Vomiting Duration: Moderate outcome-specific quality*** ^1^					
6 non-specific 3 Rotavirus	RCT	Chinese studies not placebo-controlled (−0.5)	All but one study showing decreased duration of vomiting among zinc-treated children (+1)	No non-Chinese studies (−0.5)	Generalizable
***Fever Duration: Moderate outcome-specific quality*** ^1^					
13 non-specific 4 Rotavirus	RCT	Chinese studies not placebo-controlled (−0.5)	All studies showing decreased duration of fever among zinc-treated children (+1)	No non-Chinese studies (−0.5)	Generalizable

^1^ Quality assessment scoring based on previously published criteria [11].

## 4. Discussion

The findings of our systematic review confirm and highlight the benefits of therapeutic zinc supplementation for diarrhea among children under five years of age in low- and middle-income countries. The effects of zinc treatment, which include reductions in episode duration, stool output, stool frequency and length of hospitalization, were consistent across Chinese and non-Chinese studies and non-specific and rotavirus diarrhea. These results suggest that zinc therapy of diarrhea is largely beneficial and important in both low- and middle-income settings.

The results of the large number of Chinese trials in rotavirus diarrhea are a substantial addition to the global evidence base because there have been no non-Chinese trials. One study in India based on a post-hoc subgroup analysis suggested that zinc treatment was not beneficial for rotavirus diarrhea [113]; however, the evidence from China demonstrates that therapeutic zinc supplementation reduces the duration and severity of rotavirus episodes. As rotavirus is the predominant cause of severe acute diarrhea worldwide and most likely the leading cause of diarrhea mortality [114], zinc treatment of rotavirus diarrhea could potentially yield large reductions in hospitalizations and deaths.

In comparison to non-Chinese studies, the difference between the mean episode duration of zinc-treated and control group children was statistically significantly higher for Chinese studies (*p* < 0.05). It is possible that this difference resulted from lack of placebo-controlled groups and blinding among Chinese studies. However, estimates of the effects of therapeutic zinc supplementation on other outcomes were largely consistent across study locations and we were able to generate a pooled global effect size for the proportion of episodes >3days. The consistency of effect estimates between studies conducted in and outside China suggests that the lack of placebo-controlled groups in Chinese studies did not greatly bias the results.

Zinc dose did not affect the estimate of the effect of zinc supplementation on the duration of diarrhea for non-Chinese or Chinese studies. Although Licorzinc was associated with slightly greater reductions in the mean duration of non-specific diarrhea than other zinc products, zinc effect sizes were generally comparable across Chinese studies regardless of type of zinc preparation.

There is a dearth of literature meeting our inclusion criteria that assessed diarrhea-specific and all-cause mortality. Although a previous review published mortality effect estimates [4], the sole study reporting diarrhea-specific deaths was cluster-randomized and thus violated our inclusion criteria [115]. In addition, three studies of all-cause mortality were also excluded from our review; one was on persistent diarrhea [116], and two others were review papers [3,117].

Using previously published scoring criteria, the studies included in our review yielded pooled estimates of overall moderate quality [11]. The majority of studies contributing to this review were conducted in China and South Asia; however, studies conducted outside Asia were consistent in the directionality of effect estimates. The consistency and quality of all outcomes bolsters the evidence in support of oral zinc supplementation for the treatment of acute diarrhea among children under five in low- and middle-income countries.

## 5. Conclusions

Oral therapeutic zinc supplementation reduces the morbidity of acute diarrhea among children under five in and outside China. Global efforts should be made to support scale-up of the WHO recommended regimen of therapeutic zinc in all regions.
